# Onychophagia as a clinical symptom: A pilot study of physicians and literature review

**DOI:** 10.1177/00368504211050288

**Published:** 2021-12-07

**Authors:** Sigita Lesinskiene, Kamile Pociute, Asta Dervinyte-Bongarzoni, Odeta Kinciniene

**Affiliations:** 1 Clinic of Psychiatry, Institute of Clinical Medicine, Faculty of Medicine, 54694 Vilnius University, Vilnius, Lithuania; 2 Faculty of Medicine, 54694 Vilnius University, Vilnius, Lithuania; 3 Clinic of Psychiatry, Institute of Clinical Medicine, Faculty of Medicine, 54694 Vilnius University, Vilnius, Lithuania; 4 Clinic of Children's Diseases, Institute of Clinical Medicine, Faculty of Medicine, 54694 Vilnius University, Vilnius, Lithuania

**Keywords:** Onychophagia, nail biting, mental health, physicians attitude, symptom, treatment

## Abstract

Although onychophagia is a medical condition and is associated with poorer health, there are no guidelines for assessment or treatment. The purpose of this study was to investigate the clinical aspects of nail biting from doctors’ points of view, to estimate the prevalence of onychophagia among physicians, and to review the literature on and treatment methods for onychophagia. Twenty-four percent of doctors reported nail-biting periods during their lifetimes, and 2% of them remained active nail biters. A total of 64.4% of doctors see nail biting in their practices, and 60.6% never or only on request ask patients about nail biting and examine their nails. Family doctors and pediatricians ask their patients about nail biting most often. Attitudes and opinions on the treatment of nail biting are undefined and vary. Doctors reported usually treating nail-biting patients by referring them to another specialist or offering special nail polish. There is a need to improve physicians’ knowledge of nail-biting treatment methods, but a lack of studies evaluating the clinical aspects of onychophagia and its relation to mental health and emotion dysregulation. Further research is needed. Clinical attitudes toward nail biting could be more precise in training and medical practice.

## Introduction

Onychophagia begins in childhood^1^ and over time most nail biters tend to stop biting their nails.^2^ The prevalence of nail biting in various populations ranges from 3%^3^ to 46.9%, with the highest percentage found among medical students.^2^ The etiology of onychophagia is unclear. Positive family history is found in 36.8%–63% of cases.^4,5^ No instrument has been validated to specifically measure nail biting.^6^ Studies point out that onychophagia could be classified as a tic disorder rather than obsessive-compulsive disorder.^7^ Onychophagia is associated with poorer quality of life, psychiatric disorders, higher levels of stress, social problems, and poorer physical health.^1,4,8–10^ A quarter of nail biters seek treatment, and treatment has a significant effect in reducing symptoms of onychophagia.^5^ On the other hand, 26.4% of parents do not seek treatment for onychophagia in children, and 70.2% think that punishment is an effective way to break a habit.^11^

Onychophagia could serve as an informative clinical symptom for doctors working in different fields, especially mental health. Due to the lack of scientific literature, physicians might take it upon themselves to check patients’ fingernails; results could serve as valuable clinical information and help establish a therapeutic dialogue with the patient. Having visibly bitten nails could tell both patient and doctor about the need to break the habit.

Although onychophagia is classified as a medical condition and associated with poorer health, the scientific literature lacks data on the condition from the physician's perspective. Additionally, there is little research with adults on the prevalence of nail biting. The aim of this study was to evaluate clinical aspects of onychophagia from the perspective of physicians and to determine the prevalence of onychophagia among them. A review of treatment methods was also included.

## Methods

### Participants and context

For one year in 2019, physicians at postgraduate training conferences were invited to complete a brief nail-biting questionnaire. The questionnaire was presented at an annual professional meeting, open to family doctors, pediatricians, and other medical specialists in all regions of Lithuania. Survey invitations, together with a brief description of the study, were announced during the conference. Volunteer physicians completed the questionnaires during conference breaks at a table close to the registration desk.

This pilot study was approved by the institution's ethical review board, and results are intended for use in organizing undergraduate and postgraduate training for medical doctors.

### Instrument

A 12-item, one-page nail-biting questionnaire was developed by the authors. The introductory part inquired about the respondent's age, gender, medical specialty, and years in practice. The second part was designed to cover nail biting as a symptom, and it asked physicians to indicate whether they hear complaints from patients about nail biting. If so, they were asked to write approximately how many patients complained about it over the course of a year and to note how many complaints were about nail biting in children versus adults. Physicians were also asked whether they question their patients about nail biting (*often, rarely, never, only when the patient himself has complaints*) and if they pay attention to and examine patients’ nails (*often, rarely, never, only when the patient himself has complaints)*. Respondents were asked if they ever bit their nails and if they continued to do so. The third group of questions concerned physicians’ attitudes toward treatment. Respondents were asked whether nail biting requires treatment (*does, doesn't, sometimes, I don't know*). If so, which specialists, in their opinion, should treat nail biting *(family doctor, pediatrician, psychiatrist, child and adolescent psychiatrist, psychologist, other or no treatment required*)? The questionnaire also included two open-ended questions: “What help would you offer to a nail biter?” and “Maybe you have something else to add about nail biting?”

### Statistical analysis

Data were processed using MS Excel and SPSS programs. We used descriptive statistics to describe respondent characteristics, the chi-square test for categorical variables and the Kruskal–Wallis test to compare means. A *p*-value <0.05 was considered statistically significant.

### Literature review

We decided to include studies related to the treatment of nail biting to make our research results more useful in practice. The PubMed and Cochrane Library databases were used to find studies of onychophagia treatment published between January 2013 and July 2020. The keywords used were nail biting OR onychophagia. Of the 150 studies retrieved from the PubMed database, 13 met the necessary criteria. In PubMed, we found 13 studies related to the treatment of onychophagia. Three studies were found in the Cochrane Library database, but these studies did not meet the inclusion criteria.

## Results

### Demographics and prevalence

The questionnaire was completed by 362 medical doctors, including 337 (93.0%) women and 25 (6.9%) men. A total of 123 (34.0%) respondents were family doctors, 110 (30.4%) doctors working with adult patients, and 129 (35.6%) working with children (Table 1). Median age of the survey respondents was 53.00  ±  13.994, and the median years of work experience were 25.00  ±  14.870. Eighty-six (23.8%) doctors reported nail biting during their lifetime, including 8 (2.2%) active nail biters. Eight (2.4%) doctors answered that they did not know if they bit their nails, and 2 (0.6%) did not answer. A total of 25.0% (82/328) of females and 16.0% (4/25) of men reported ever biting their nails, and there were no statistically significant differences between groups (*p* < 0.05). A total of 7.4% (6/81) of women and 25% (1/4) of men reported being current nail biters, and there were no statistically significant differences between men and women. The median age of active nail biters was 52  ±  14.547, and the median years of work experience were 27.00  ±  15.731.

**Table 1.** The specialties of physicians.

Family doctors 123 (34.0%), median age 40 ± 12.926								
Doctors working with adult patients 110 (30.4%), median age 56 ± 13.266								
Internal medicine, *n* = 27	Anesthesiology and reanimatology, *n* = 13	Physical medicine and rehabilitation, *n* = 12	Medical doctor without residency, *n* = 9	Sports medicine, *n* = 5	Psychiatry, *n* = 5	Obstetrics and gynecology, *n* = 5	Ophthalmology, *n* = 4	Forensic medicine, *n* = 3
Nephrology, *n* = 3	Otorhinolaryngology, *n* = 3	Surgery, *n* = 3	Cardiology, *n* = 2	Geriatrics, *n* = 2	Radiology, *n* = 2	Allergology and clinical immunology, *n* = 1	Dietology, *n* = 1	Endocrinology, *n* = 1
Endoscopy, *n* = 1	Gastroenterology, *n* = 1	Infectology, *n* = 1	Neurology, *n* = 1	Orthopedics and traumatology, *n* = 1	Proctology, *n* = 1	Psychotherapy, *n* = 1	Rheumatology, *n* = 1	Urology, *n* = 1
Doctors working with children 129 (35.6%), median age 56 ± 12.847								
Pediatrics, *n* = 86	Cardiology, *n* = 13	Neonatology, *n* = 12	Neurology, *n* = 3	Ophthalmology, *n* = 3	Endocrinology, *n* = 2	Intensive therapy, *n* = 2	Pulmonology, *n* = 2	Allergology and clinical immunology, *n* = 1
Children and adolescent psychiatry, *n* = 1	Gastroenterology, *n* = 1	Nephrology, *n* = 1	Rheumatology, *n* = 1	Surgery, *n* = 1				

### Clinical practice

According to respondents, 233 (64.4%) had heard complaints about nail biting in their practices. On average, 5.2 patients (4.5 children and 0.7 adults) complained of onychophagia per year. Statistically significant, most complaints about nail biting were received by children's doctors (Table 2).

**Table 2.** Means of patient complaints per year.

	*N* (%)	Total	About children	About adults
Family doctors	110 (35.83%)	4.5	3.5	1.0
Working with adults	88 (28.66%)	2.8	2.0	0.8
Working with children	109 (35.50%)	7.8	7.5	0.3
Total	307 (100%)	5.2	4.5	0.7
Sig.		0.000	0.000	0.000

ANOVA, analysis of variance.

*P* values reflect the results of the Kruskal–Wallis 1-way ANOVA test to compare means.

A total of 212 (60.6%) of 350 doctors never asked patients about nail biting, 118 (32.6%) rarely asked them, and 20 (5.5%) often asked them. Most physicians (60/17.6%) reported never (or only when the patient complained) paying attention and examining patients’ nails: 100 (29.4%) rarely, and 180 (52.9%) often. We categorized “often” and “rarely” as “initiate” and “never,” respectively, and “only when the patient complains” as “do not initiate.” We found (using chi-square test) that data were statistically significant among types of physicians, *p*  =  0.000 (*p* < 0.05), but we found no statistical significance among groups regarding examination of patients’ nails, *p* = 0.237 (*p* > 0.05) (Table 3 and Table 4).

**Table 3.** Frequency of asking about nail biting.

	Family doctors (*N* = 121) *n* (%)	Adult's doctors (*N* = 107) *n* (%)	Children's doctors (*N* = 122) *n* (%)	*p*
Often	5 (4.1%)	2 (1.9%)	13 (10.7%)	0.000
Rarely	49 (40.5%)	22 (20.6%)	47 (38.5%)	
Never	32 (26.4%)	53 (49.5%)	23 (18.9%)	
Only when the patient has complaints	35 (28.9%)	30 (28.0%)	39 (32.0%)	
Initiate (often + rarely)	54 (44.6%)	24 (22.5%)	60 (49.2%)	0.000
Do not initiate (never + only when the patient has complaints)	67 (55.3%)	83 (77.5%)	62 (50.9%)	

*P* values reflect the results of the chi-square test comparing categorical variables.

**Table 4.** Frequency of patients’ nail examination.

	Family doctors, *n* (%)	Adult's doctors, *n* (%)	Children's doctors, *n* (%)	*p*
Often	61 (50.8%)	37 (36.6%)	82 (68.9%)	0.000
Rarely	42 (35.0%)	41 (40.6%)	14 (14.3%)	
Never	2 (1.7%)	11 (10.9%)	4 (3.4%)	
Only when the patient has complaints	15 (12.5%)	12 (11.9%)	16 (13.4%)	
Initiate (often + rarely)	103 (85.8%)	78 (77.2%)	96 (83.2%)	0.237
Do not initiate (never + only when the patient has complaints)	17 (14.2%)	23 (22.8%)	20 (16.8%)	

*P* values reflect the results of the chi-square test comparing categorical variables.

### Treatment

Regarding the belief that nail biting needs treatment, 266 (73.5%) of 358 of doctors said yes, 18 (5.0%) said no, 39 (10.8%) reported that nail biting should be treated sometimes, and 35 (9.7%) said they did not know.

Three-quarters of respondents (250/75%) answered the open-ended question, “What help would you offer to a nail-biter”? The most common (∼50%) response was referral to another specialist, 82 respondents said that they would refer the patient to a psychologist, 24 to a psychotherapist, 13 to a psychiatrist, 7 to a neurologist, a dentist, and an orthopedist were mentioned once. A few of the others noted “doctor” or “specialist,” without naming a specialization.

The second most common (∼25%) was to provide a special nail polish. Several respondents said that it would be important to discover the reason for onychophagia. Some said that it would be helpful to distract a child with another activity.

When was asked to choose which specialist should treat nail biting, 69.9% of respondents said a psychologist should treat nail biting, 45.3% a family doctor, 43.6% a child/adolescent psychiatrist, 36.7% a pediatrician, 29.6% a psychiatrist, 8.8% other, and 3.3% of respondents noted that treatment was unnecessary.

There were no statistically significant differences between the physicians who bit their nails and those who did not on any treatment choices.

## Literature review

### Therapy

Research has shown that cognitive psychophysiological treatment after 14 weeks of therapy reduces symptoms of body-focused repetitive behaviors, such as nail biting, hair pulling, and skin picking. In this study, 54 participants completed the program, 22 of whom engaged in nail biting. Seventy-four percent of all patients showed significant improvement, as well as improved mood and self-esteem. After 6 months of follow-up, the decrease in symptoms was maintained.^7^

A randomized controlled trial reported that habit reversal training (HRT) and object manipulation training (OMT) could be useful in reducing symptoms of chronic nail biting in children and adolescents. Subjects were 91 children randomly assigned to three groups. After three months of therapy, the mean length of the nails in the HRT and OMT groups increased significantly compared to the control group, and HRT was more effective than OMT.^12^

The case report of a 4-year-old boy diagnosed with autism spectrum disorder showed a significant effect of differential reinforcement of other behavior in reducing nail biting at near-zero levels.^13^

A habit reversal treatment in combination with auricular acupressure was found more effective than habit reversal treatment in combination with placebo auricular acupressure for treating nail biting.^14^

### Pharmacotherapy

A 24-year-old male with long-lasting onychophagia was successfully treated with *N*-acetylcysteine.^15^ However, one randomized controlled trial failed to find a statistically significant nail length difference in children and adolescents (*n* = 42) with chronic nail biting following the administration of *N*-acetylcysteine (800 mg/day) versus placebo after two months.^16^

When lithium was given to a 28-year-old woman with a bipolar II disorder and in remission for substance use disorder, she stopped biting her nails, even though the habit had bothered her since the age of 12, and various other methods, such as HRT, self-monitoring, and competing response, had been ineffective.^17^

Milk thistle (150 mg/day) was effective for a 24-year-old woman who had bitten her nails since age 7. Nail biting resumed after discontinuation of treatment, but the women stopped nail biting again after 4 weeks of resuming the food supplement.^18^

### Topical intervention

Research with 80 nail biters showed that nonremovable reminder vinyl wristbands and bitter-tasting nail enamel were effective in reducing nail biting, while nail enamel was found to be more effective than wristbands.^19^

A case report revealed that a special dental appliance that mechanically stopped the action of onychophagia and was maintained for one month was effective for a 26-year-old male. During the 9-month follow-up, the patient reported that the treatment met his expectations.^20^ Another case report of an 11-year-old boy confirmed the success of the dental appliance.^21^

### Special programs in school

Two studies investigating complex programs presented in schools. “Do Not Bite Your Nails, Cut Your Nails,”^11^ and *the healthy nails program*,^22^ designed to reduce nail biting in children, have shown that such programs are an effective way to reduce onychophagia.

## Discussion and conclusions

A quarter of physicians surveyed reported that they have bitten their nails, and this number corresponds to the general prevalence of onychophagia found in the literature. Interestingly, the prevalence found in our study is twice as low as that in the study by Pacan et al., which investigated nail-biting prevalence among medical students.^2^ About 2% of physicians in our study continued to bite their nails, which is quite a small percentage compared to the literature. However, it makes sense. Our study and the literature review show that the symptoms of onychophagia decrease with age, and our study looked at the oldest population found in the literature. In contrast, most researchers study nail biting in children. Our study found that women more often bite their nails, but this difference was not statistically significant. The data found in the literature are mixed. Some studies indicate that there is no significant difference between the genders^5,11,23^; others show that women bite their nails more often than men.^3,6^

More than half of all doctors have heard complaints about nail biting from patients. Most complaints of nail biting are heard by doctors working with children, and the fewest complaints about nail biting are heard by adult doctors; they are also the least likely to ask about nail biting. Three-quarters of doctors believe that nail biting should be treated, but they themselves rarely ask about nail biting. When asked “What help would you offer to a nail biter?” The most frequent answer was referral to a specialist, and the second was to recommend a bitter-tasting nail polish. Nearly 70% of physicians believe that nail biting should be treated by a psychologist. Interestingly, the answers to questions about the need for treatment are different; individuals changed their minds when filling in the questionnaire, and more of them decided that nail biting should be treated. Only 5 of 18, who initially (in question 8) marked that no treatment is needed for onychophagia, stayed with their opinions (in question 12). It is possible that completing the questionnaire increased the importance of nail nail-biting treatment for the respondents. Our study showed that nail-biting habits possibly do not change an individual's perspective on the necessity for treatment or treatment methods. Onychophagia can be an indicator of relatively poor physical and mental health; furthermore, patients with more severe forms differ from subclinical forms and are in poorer health.^24^ There are ways to help reduce the symptoms of onychophagia. Unfortunately, there is a lack of strong evidence for various methods that could help, but the literature describes some therapeutic and pharmacotherapeutic methods and other techniques that can reduce nail biting. How onychophagia is related to mental health disorders, anxiety, impulsivity, self-regulatory behavior, and emotion dysregulation remains unclear.

Although onychophagia is associated with poorer health, on average, less than half of nail biters seek help. Developing and implementing psychoeducational programs in schools to teach both children and adults about nail biting has proven effective in reducing the symptoms of onychophagia. The importance of psychoeducational programs was also demonstrated by a study that found most parents (91%) have punished their children at least once for biting their nails.^11^ There is still a lack of high-quality research evaluating the treatment methods and other clinical aspects of onychophagia, so further research is needed.

This pilot study revealed that clinical attitudes toward nail-biting symptoms could be more precise in medical practice and training. It is important to increase physicians’ knowledge about nail-biting treatment methods. Developing a validated tool to assess nail biting would be useful in both clinical practice and research. Scale for anxiousness and emotion dysregulation could be elaborated and used simultaneously. Finally, developing an algorithm for the treatment of onychophagia that informs physicians about how to evaluate onychophagia, when to refer a patient to another specialist, and how to treat onychophagia themselves. Cross-cultural studies and further research in this area are needed.
